# Associations between ambient air pollutants and childhood hand, foot, and mouth disease in Sichuan, China: a spatiotemporal study

**DOI:** 10.1038/s41598-023-31035-7

**Published:** 2023-03-10

**Authors:** Jian Qian, Caiying Luo, Qiang Lv, Yaqiong Liu, Tao Zhang, Fei Yin, Yue Ma, Tiejun Shui

**Affiliations:** 1 West China School of Public Health and West China Fourth Hospital, Sichuan University, Chengdu, Sichuan China; 2 Sichuan Center for Disease Control and Prevention, Chengdu, Sichuan China; 3 Yunnan Center for Disease Control and Prevention, Kunming, Yunnan China

**Keywords:** Environmental impact, Risk factors, Infectious diseases

## Abstract

Hand, foot, and mouth disease (HFMD) is a major public health concern in the Asia–Pacific region. Previous studies have implied that ambient air pollution may affect the incidence of HFMD, but the results among different regions are inconsistent. We aimed to deepen the understanding of the associations between air pollutants and HFMD by conducting a multicity study. Daily data on childhood HFMD counts and meteorological and ambient air pollution (PM_2.5_, PM_10_, NO_2_, CO, O_3_, and SO_2_) concentrations in 21 cities in Sichuan Province from 2015 to 2017 were collected. A spatiotemporal Bayesian hierarchical model framework was established, and then a distributed lag nonlinear models (DLNMs) was constructed to reveal exposure-lag-response relationships between air pollutants and HFMD while controlling for spatiotemporal effects. Furthermore, given the differences in the levels and seasonal trends of air pollutants between the basin region and plateau region, we explored whether these associations varied between different areas (basin and plateau). The associations between air pollutants and HFMD were nonlinear, with different lag responses. Low NO_2_ concentrations and both low and high PM_2.5_ and PM_10_ concentrations were associated with a decreased risk of HFMD. No significant associations between CO, O_3_, and SO_2_ and HFMD were found. The associations between air pollutant concentrations and HFMD were different between the basin and plateau regions. Our study revealed associations between PM_2.5_, PM_10_, and NO_2_ concentrations and HFMD, deepening the understanding of the relationships between air pollutants and HFMD. These findings provide evidence to support the formulation of relevant prevention measures and the establishment of an early warning system.

## Introduction

Hand, foot, and mouth disease (HFMD), a worldwide public health issue, has become a predominant communicable disease in the Asia–Pacific region during the last two decades^1,2^. With the highest annual number of disability-adjusted life-years (96 900) in the world and more than one million cases reported each year, China has had one of the highest HFMD disease burdens since 2008^3–5^. HFMD is an acute infection in childhood caused by various enteroviruses^6^. Although most patients experience only mild clinical manifestations, some patients may develop severe neurological complications and even die^7^. To date, there is no specific antiviral treatment for HFMD, and licensed vaccines are effective for only enterovirus 71^8^. Hence, preventing HFMD with public health-related measures is important. Considering the substantial threat to child health, identifying risk factors is urgent for the prevention of HFMD.

The adverse health effects associated with ambient air pollution have prompted increasing concern in recent decades due to its substantial contribution to the global burden of disease^9^. Recently, some studies have implied that ambient air pollution may affect the incidence of HFMD^10–17^. Most previous studies employed time-series analyses, such as distributed lag nonlinear models (DLNMs), according to the nonlinear and lagged associations between air pollutant concentrations and HFMD^18^. However, such studies were carried out in single cities, and the associations among different regions were inconsistent in shape and magnitude. For example, Gu et al*.* indicated an approximately J-shaped association between the NO_2_ concentration and HFMD in Ningbo city, whereas Liu et al*.* found an inverted V-shaped association in Shijiazhuang city^12,13^. A study in Guilin city showed that a low PM_2.5_ concentration (1st vs. median) decreased the risk of HFMD, while another study in Shenzhen city found no significant association between PM_2.5_ concentration and HFMD^15,16^. Such inconsistencies indicate spatial heterogeneity among different regions, especially regions with different socioeconomic characteristics and air pollution levels. Hence, to attain a more comprehensive understanding of the associations between air pollution concentrations and HFMD, a study accounting for spatial heterogeneity should be carried out in multiple locations. To our knowledge, only one published study on the associations between air pollutant concentrations and HFMD in Shenzhen city accounted for such spatial effects with a spatiotemporal Bayesian model^19^. However, this study did not consider the nonlinear and lag associations between air pollution concentrations and HFMD. Moreover, the between-site variability in Shenzhen city was limited. To reveal the nonlinear and lag associations, the DLNM can be introduced into the spatiotemporal Bayesian hierarchical model, which simultaneously assesses exposure- and lag-response associations between air pollutants and HFMD with adjustments for spatiotemporal effects^20,21^.

Sichuan Province has a high HFMD disease burden, with a total of 449 485 HFMD cases reported from 2011 to 2017^22^. Furthermore, Sichuan Province is one of China’s most air-polluted areas^23^. Therefore, to reveal more comprehensive and general associations of air pollutant concentrations with HFMD, we conducted a spatiotemporal study in 21 cities in Sichuan Province. Specifically, we employed a spatiotemporal Bayesian hierarchical model framework based on the daily number of pediatric HFMD cases in 21 cities in Sichuan Province from 2015 to 2017. By incorporating the DLNM, we assessed the exposure-lag-response associations of air pollutants with HFMD after controlling for spatiotemporal effects. Furthermore, given the differences in the levels and seasonal trends of air pollution between the basin region and plateau region in Sichuan Province, we explored whether the associations between air pollutant concentrations and HFMD varied between the different areas (basin and plateau).

## Results

A total of 214 059 HFMD cases in patients aged 0–14 years were reported in Sichuan Province from 2015 to 2017. Table 1 shows the characteristics of the daily HFMD cases, air pollutants, and meteorological variables. Sichuan Province has serious air pollution contamination, especially PM and NO_2_ contamination. On 89%, 68%, and 59% of the days, the concentrations of PM_2.5_, PM_10_, and NO_2_ exceeded the values recommended by the World Health Organization, respectively (WHO 2021). The concentration of SO_2_ in the plateau region was higher than that in the basin region, while the concentrations of PM_2.5_ and PM_10_ were much lower in the plateau region (Table 1).

**Table 1:** Descriptions of daily HFMD cases, air pollutants, and meteorological variables in Sichuan Province from 2015 to 2017.

Variable	Mean	SD	*P*_1_	*P*_25_	Median	*P*_75_	*P*_99_	Range
HFMD (cases)	9.3	19.7	0.0	2.0	4.0	9.0	117.0	(0.0, 268.0)
Basin	10.8	21.4	0.0	2.0	5.0	10.0	125.0	(0.0, 268.0)
Plateau	3.0	4.9	0.0	0.0	1.0	4.0	28.0	(0.0, 49.0)
PM_2.5_ (μg/m^3^)	44.8	34.2	6.0	21.0	35.0	58.0	167.0	(2.0, 370.0)
Basin	49.9	35.7	7.0	25.0	40.0	65.0	173.0	(2.0, 370.0)
Plateau	20.2	10.0	4.0	13.0	18.0	25.0	61.0	(3.0, 105.0)
PM_10_ (μg/m^3^)	72.8	47.5	14.0	39.0	60.0	93.0	236.0	(3.0, 599.0)
Basin	79.8	49.1	16.0	45.0	68.0	103.0	243.0	(6.0, 599.0)
Plateau	43.0	22.4	10.0	27.0	38.0	54.0	114.0	(3.0, 142.0)
NO_2_ (μg/m^3^)	29.1	14.0	6.0	20.0	27.0	36.0	68.0	(2.0, 471.0)
Basin	30.5	12.7	10.0	21.0	29.0	38.0	69.0	(4.0, 112.0)
Plateau	23.1	17.4	5.0	13.0	21.0	29.0	62.0	(2.0, 471.0)
CO (mg/m^3^)	0.9	0.4	0.3	0.6	0.8	1.1	2.1	(0.1, 4.0)
Basin	0.9	0.3	0.3	0.6	0.8	0.9	1.9	(0.1, 4.0)
Plateau	0.9	0.5	0.2	0.5	0.9	1.2	2.6	(0.1 2.0)
O_3_ (μg/m^3^)	62.3	33.6	10.0	38.0	57.0	80.0	169	(2.0, 243.0)
Basin	62.4	35.0	10.0	37.9	56.0	81.0	174.0	(2.0, 242.0)
Plateau	61.9	26.8	17.0	42.0	59.0	77.0	140.0	(5.0, 187.0)
SO_2_ (μg/m^3^)	15.8	10.2	3.0	9.0	13.0	20.0	54.0	(1.0, 117.0)
Basin	14.5	8.5	3.0	9.0	13.0	18.0	44.0	(1.0, 112.0)
Plateau	21.4	14.4	3.0	11.0	18.0	29.0	71.0	(2.0, 117.0)
Temperature (℃)	16.5	8.0	− 3.7	10.0	16.8	22.9	31.3	(− 16.2, 34.3)
Humidity (%)	73.8	14.0	30.8	66.3	75.3	84.1	97.0	(12.7, 101.3)

P1, p25, p75, and p99 represent the 1st, 25th, 75th, and 99th percentiles of each variable, respectively.

**Figure 1:**
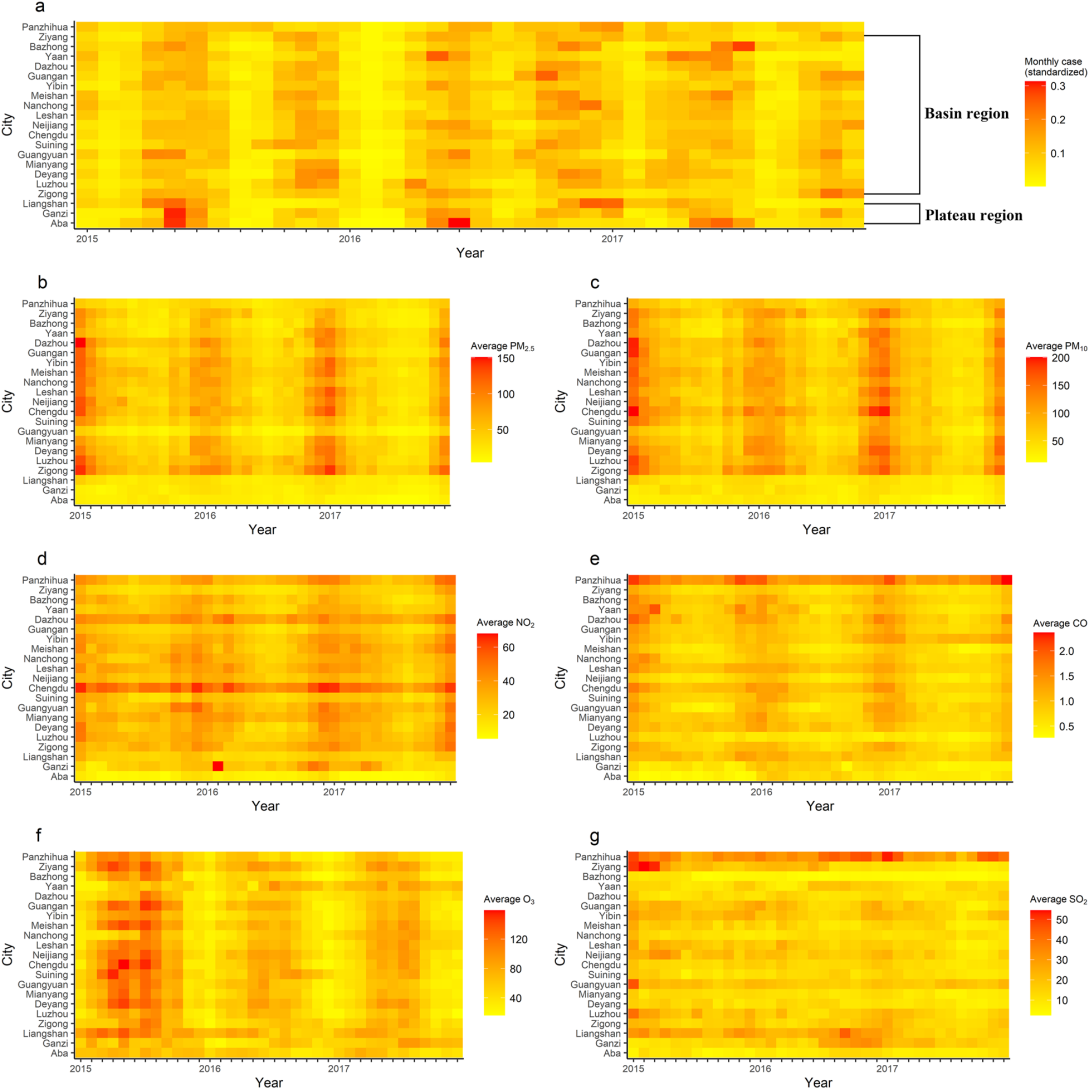
Heatmaps of (**a**) monthly HFMD cases (standardized by the number of annual cases), (**b**) average PM_2.5_, (**c**) average PM_10_, (**d**) average NO_2,_ (**e**) average CO, (**f**) average O_3_, and (**g**) average SO_2_ concentrations. The figure was generated by the R software version 4.0.3 (https://www.r-project.org/).

**Figure 2:**
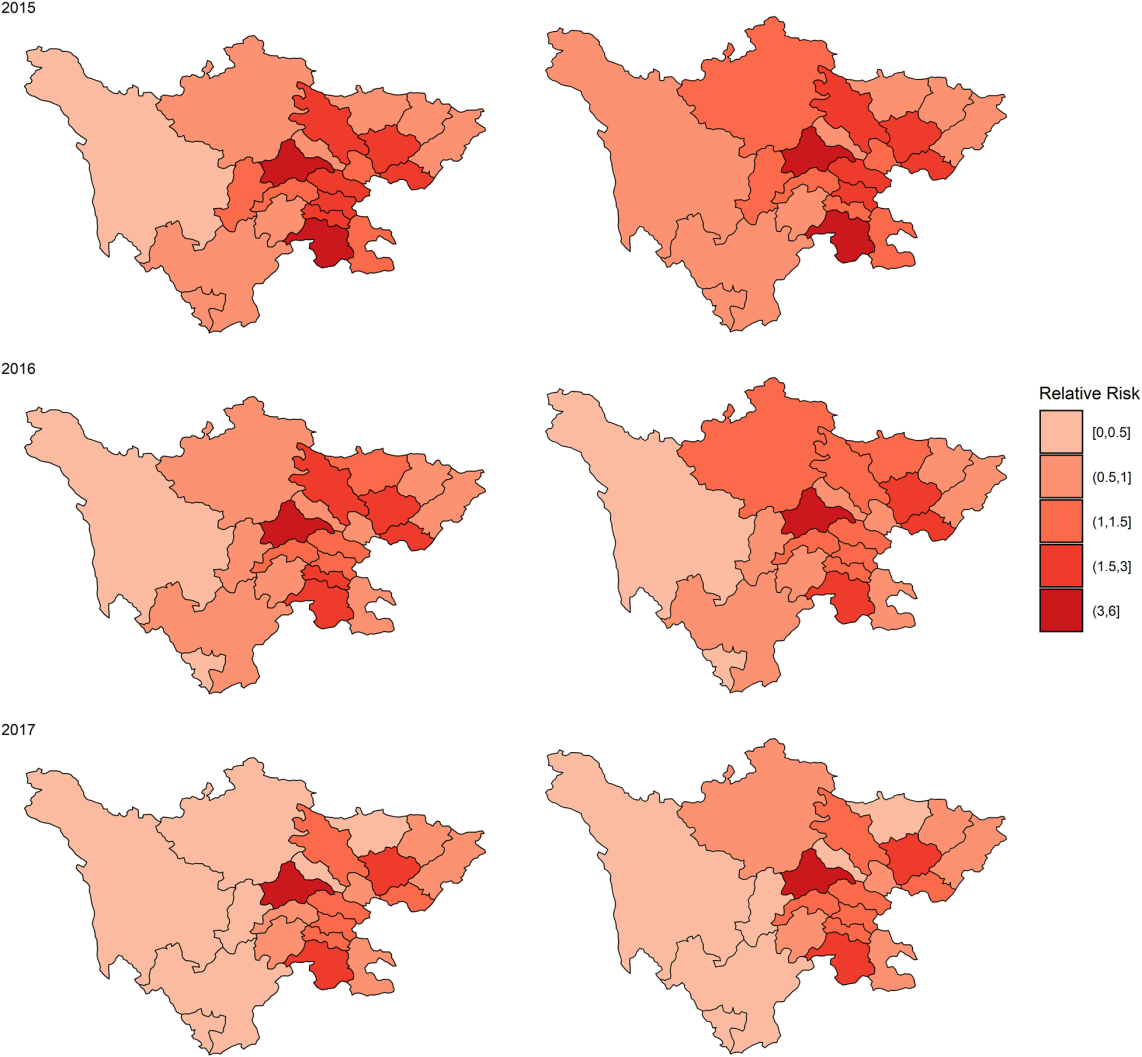
Annual RRs of HFMD in 21 cities in Sichuan from 2015 to 2017 by the model without covariables (left) and the model with air pollutants (right).

Monthly HFMD cases are presented in Fig. 1a. The number of HFMD cases in Sichuan Province exhibited a seasonal trend with two peaks per year: one from approximately April to July and the other from approximately October to December. The monthly distributions of air pollutants are illustrated in Fig. 1b–f. PM_2.5_, PM_10_, NO_2_, and CO concentrations had similar trends, with higher values in winter and lower values in summer (Fig. 1b–e), while O_3_ presented the opposite trend (Fig. 1f). No distinct trend was observed for SO_2_ (Fig. 1g). In addition, PM_2.5_, PM_10_, and NO_2_ concentrations in the plateau region presented no significant seasonal trends. Figure 2 (left) shows the annual RRs of HFMD for the 21 cities from 2015 to 2017. We obtained these estimations by fitting the spatiotemporal model without explanatory variables. Overall, the region with the highest risk of HFMD was the central region of Sichuan Province, and the highest RR was found in Chengdu City in 2015 (5.16, 95%CI 4.13–7.64). Figure 2 (right) displays the annual RRs with the air pollutants included in the model. These results indicate that most cities still have an excessive risk of HFMD, which cannot be explained by air pollution. But the RR of HFMD has also changed in some cities, for example, the RR of HFMD in Aba Prefecture in 2015 changed from 0.50 (95%CI 0.29–0.88) to 0.99 (95%CI 0.57–1.73) after including air pollutants.

**Figure 3:**
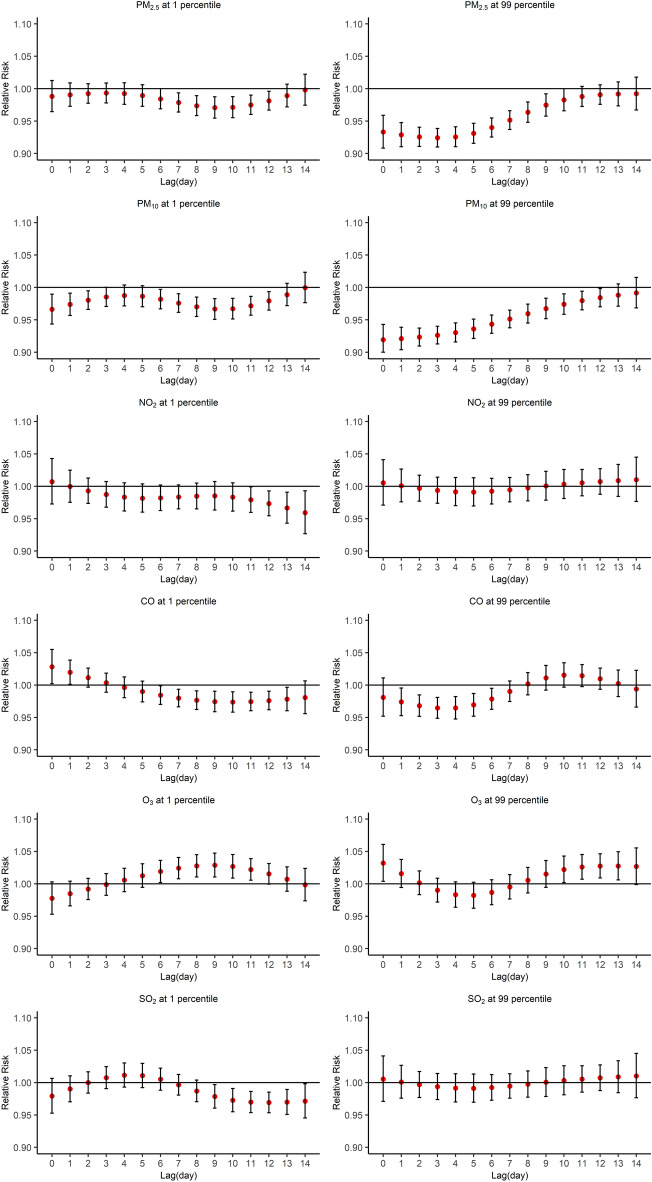
Associations between air pollutants at the 1st (left) and 99th (right) percentiles and HFMD across lag days.

According to the DIC results, the ${df}_{l}$ in the lag dimension for all six air pollutants were set to 3. And the ${df}_{v}$ in the exposure dimension for O_3_ and PM_2.5_ were set to 4, while the other air pollutants were set to 3 (Supplementary Tables S1 and S2). Figure 3 shows the associations of air pollutants at the 1st and 99th percentiles with HFDM across the lag period. These effects of low and high concentrations of air pollutants on HFMD on different lag days mainly decrease the relative risk. The most significant negative effects are the 99th percentiles of PM_2.5_ and PM_10_ during 0–9 lag days and 0–11 lag days, respectively. And the lowest relative risks of the 99th percentiles of PM_2.5_ and PM_10_ are 0.92 (95%CI 0.91–0.94) and 0.92 (95%CI 0.90–0.94) on lag 3 day and lag 0 day, respectively. However, the 1st percentile of O_3_ increases the relative risk during 6–11 lag days, the highest relative risk is 1.03 (95CI 1.01–1.05) on lag 9 day. Besides, only the 99th percentiles of SO_2_ and NO_2_ found no significant associations with HFMD across the entire 0–14 days lag period. Supplementary Fig. S1 indicates the exposure-lag-response relationships between air pollutants and HFMD during the 0–14 days lag period. Overall, the associations of air pollutant concentrations with HFMD were nonlinear, and the lag structures were markedly different. For instance, a low PM_10_ (32 μg/m^3^) concentration decreased the risk of HFMD over the 0–14 days lag period, while a high PM_10_ concentration increased the risk of HFMD at lag 13–14 days.

**Figure 4:**
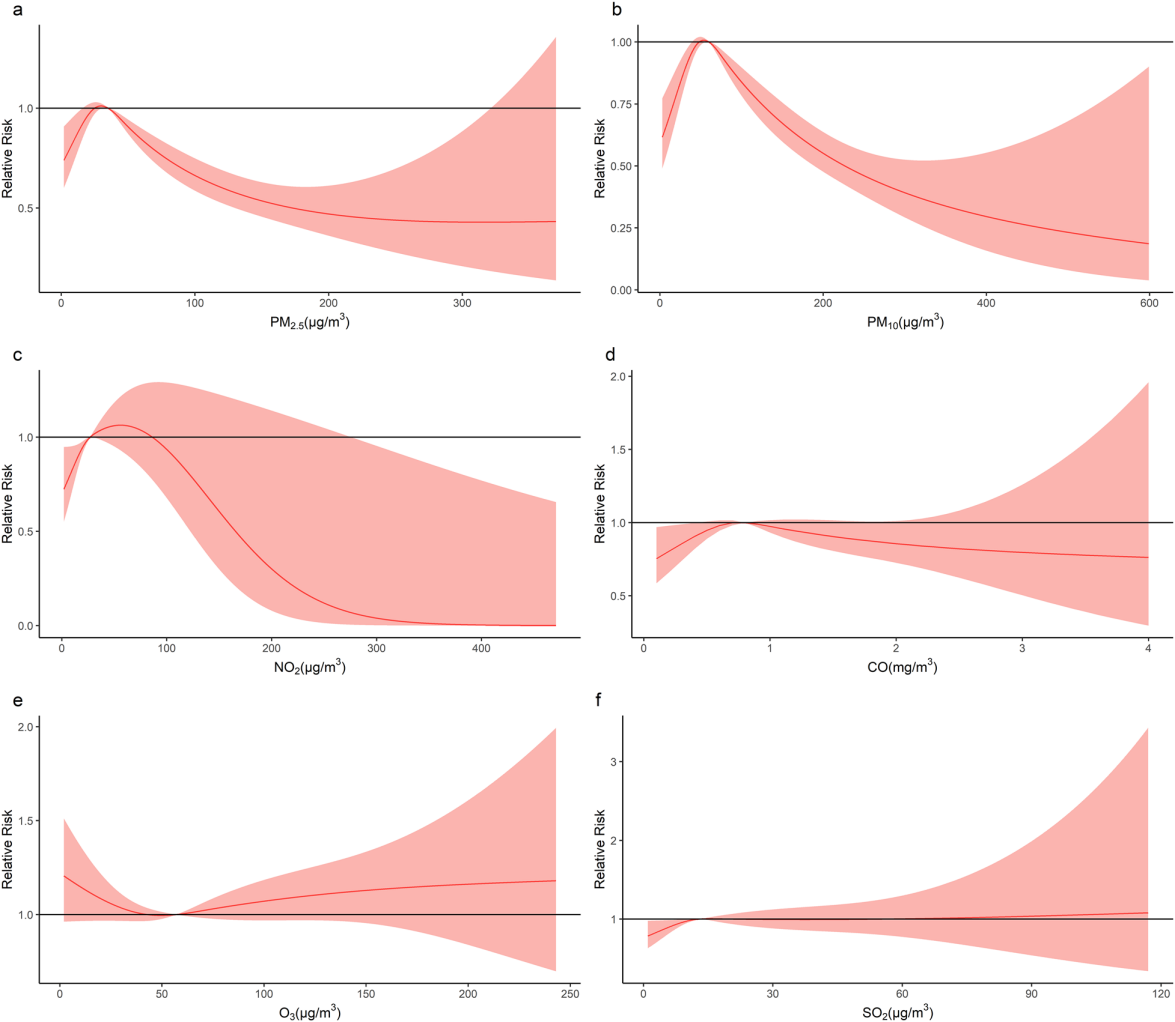
Cumulative associations of air pollutants with HFMD.

Figure 4 illustrates the cumulative association of air pollutant concentrations with HFMD over the 14 days lag period, and the median of each air pollutant was selected as the reference value. The results indicated similar associations between PM_2.5_ and PM_10_, and HFMD, with approximately inverted V-shaped curves (Fig. 4a,b). The cumulative RR initially increased with increasing air pollutant concentrations and then decreased, peaking at 29.7 μg/m^3^ and 54.6 μg/m^3^, respectively. A low NO_2_ concentration (< 50th percentile) showed a positive association with HFMD (Fig. 4c). No significant associations were found between CO, O_3_ and SO_2_ and HFMD.

**Figure 5:**
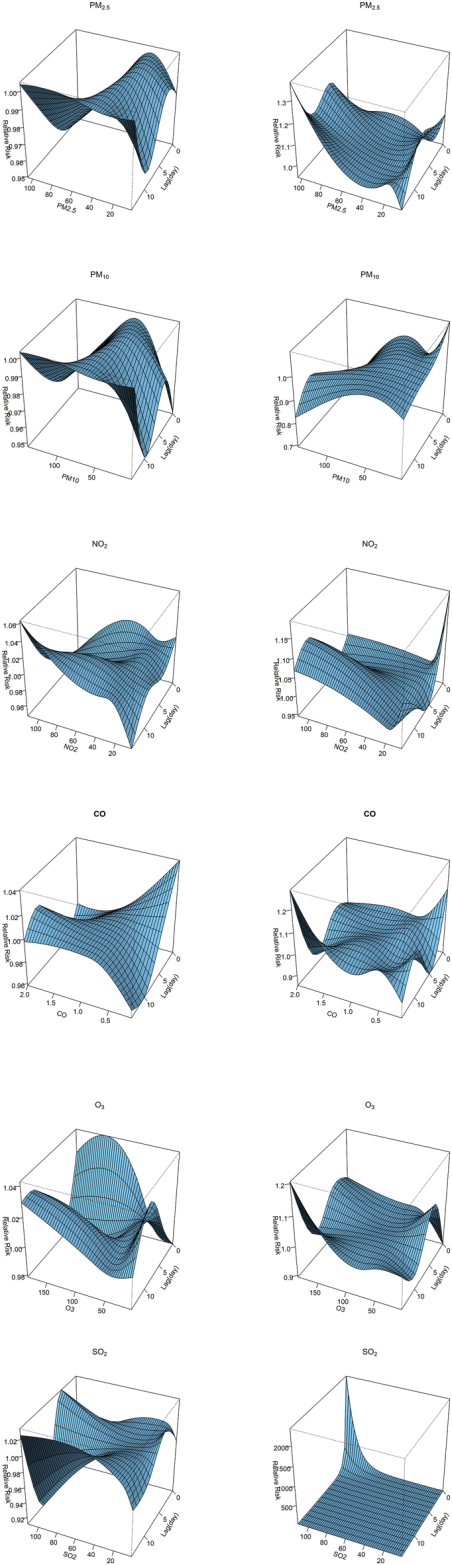
Relationships between air pollutants and HFMD over the 0–14 days lag period in the basin region (left) and plateau region (right).

Figure 5 displays the exposure-lag-response associations of air pollutants with HFMD in the basin region and plateau region (Liangshan, Ganzi, Aba) of Sichuan Province. Both the exposure–response and lag-response relationships between air pollutants and HFMD among the two regions were markedly different. For example, a high PM_2.5_ concentration (105 μg/m^3^) was associated with a decreased risk (RR = 0.98, 95%CI 0.97–0.99) of HFMD at lag day 10 in the basin region. In the plateau region, a high PM_25_ concentration (105 μg/m^3^) was associated with an increased risk (RR = 1.16, 95%CI 1.03–1.34) of HFMD at lag day 10.

The results of the sensitivity analyses were presented in the Supplementary Information.

## Discussion

In this study, we conducted a spatiotemporal analysis in 21 cities in Sichuan Province. By introducing the DLNM into the spatiotemporal Bayesian hierarchical model framework, we assessed the exposure- and lag-response associations of air pollutants with HFMD after adjustments for spatiotemporal effects, which revealed a more general association between air pollutant concentrations and HFMD. The introduction of spatial effects in this study allowed a more precise estimation of the associations than could be obtained with two-stage time-series analysis^24^, which is usually employed to pool multilocation estimations (Supplementary Fig. S2). The cumulative exposure–response associations indicated that low NO_2_ and both low and high PM_2.5_ and PM_10_ concentrations were associated with a decreased risk of HFMD. However, no significant associations between CO, O_3_ and SO_2_ concentrations and HFMD were observed.

The exact mechanisms of these associations remain unclear to date, but the effect of air pollutants on HFMD transmission may be due to the fact that air pollutants change people’s behavior and their susceptibility to enteroviruses. A reasonable explanation for the inverted V-shaped associations of PM_2.5_ and PM_10_ with HFMD is that particles mater may initially increase the risk of HFMD by promoting viral transmission or increasing HFMD susceptibility in children, which has been observed in previous studies^13,16^. Studies have found that enteroviruses may attach to particles mater in the air that can be inhaled; thus, increasing concentrations of PM_2.5_ and PM_10_ can increase the risk of HFMD by facilitating transmission^25,26^. In addition, exposure to fine particles mater triggers an inflammatory response in the body, which may increase the susceptibility of children to HFMD pathogens^27^. However, people may change their behaviors on days with high concentrations of PM_2.5_ and PM_10,_ which decreases the risk of contracting HFMD. For example, on days with serious air pollution, people may alter their behaviors by reducing the time spent performing outdoor activities or wearing masks, which could decrease the risk of HFMD^28,29^. Moreover, given that PM_2.5_ and PM_10_ are the main air pollutants in the study region, the air quality index value referenced by the local government when issuing air quality warnings mainly reflects the concentrations of PM; thus, the associations of high concentrations of PM_2.5_ and PM_10_ were significant. Regarding the association of a low NO_2_ concentration with an increased risk of HFMD, previous studies found that exposure to NO_2_ may increase the risk of acute respiratory outcomes, such as upper respiratory tract infection and impaired lung function^30,31^. Therefore, NO_2_ may increase HFMD susceptibility in children by damaging the respiratory system. However, these hypotheses need to be validated in the future.

Since the spatiotemporal Bayesian hierarchical model was constructed to investigate the associations of air pollutants with HFMD in our study, city-specific socioeconomic characteristics, such as healthcare access, were adjusted by spatial random effects. Hence, some of the differences in the results between our study and other single-city studies may be attributed to specific socioeconomic characteristics in other study regions. In addition, differences in air pollution levels among different regions could also be a possible reason for these inconsistent associations. For instance, a study in Shenzhen city indicated no significant association of the PM_2.5_ concentration with HFMD^15^. A possible reason is that the mean value of PM_2.5_ in Shenzhen was much lower than that in Sichuan (32.1 μg/m^3^ vs. 44.8 μg/m^3^). Several previous studies also indicated that the adverse health impacts of air pollutants varied among regions with different air pollution levels^32,33^. Similarly, a study in Ningbo city found a J-shaped relationship between the NO_2_ concentration and HFMD, and the mean NO_2_ concentration in Ningbo was much lower than that in Sichuan (15.2 μg/m^3^ vs. 29.1 μg/m^3^)^12^. Another study in Shijiazhuang city, which is heavily polluted by NO_2_, found an inverted V-shaped curve, similar to the results in our study^13^. For SO_2_ and O_3_ concentrations, Gu et al. found J-shaped and V-shaped associations with HFMD in Ningbo city, respectively^12^. However, SO_2_ and O_3_ pollution was not serious in Sichuan Province, and the imprecise estimations of high SO_2_ and O_3_ concentrations indicated no significant associations. Another possible explanation for the differences in associations could be the seasonal trends of air pollutants. For example, a study in Ningbo city showed no significant association of the PM_10_ concentration with HFMD^34^. Although PM_10_ pollution was heavy in Ningbo, no obvious seasonal PM_10_ trends were found in Ningbo, which is an industrial city. No significant association between the CO concentration and HFMD was found in our study, which is consistent with the results of previous studies^15,35^.

As expected, the results from the basin region and plateau region were substantially different. There are some possible reasons for these inconsistent results. First, the different associations of PM_2.5_, PM_10_, NO_2_, and SO_2_ with HFMD may be caused by different concentration levels between the two regions. The pollution levels of PM_2.5_, PM_10_, and NO_2_ in the basin region were higher than those in the plateau region, while SO_2_ showed the opposite trend. Second, some studies have suggested that the relationships between air pollutants and HFMD may be affected by seasons, and the effects of air pollutants during the cold season are greater than those during the warm season^10,17^. Therefore, differences in the seasonal trends of air pollutants concentrations may cause different relationships between air pollutants and HFMD. Compared to those in the basin region, PM_2.5_, PM_10_, NO_2_, CO, and O_3_ concentrations showed no significant seasonal trends in the plateau region, which could be a reason for these inconsistent results. In addition, the associations between air pollutants and HFMD may be modified by terrain and climate factors, but investigating these modifying effects was beyond the scope of our study. However, given that there were only three cities in the plateau region, the precision of these estimations needs to be improved. Further studies including more cities in the plateau region are needed.

There are some limitations in this study that should be acknowledged. First, CO, O_3_, and SO_2_ pollution was not serious in Sichuan Province, which prevented us from revealing associations between these air pollutants and HFMD under high-pollution conditions. Further investigations are needed in regions with high levels of CO, O_3_, and SO_2_. Second, this study was carried out in 21 cities in Sichuan Province, which is located in southwest China. Thus, these results cannot be generalized to other areas with different air pollution levels. More investigations are needed in various geographical areas and at larger spatial scales in the future. Third, some important factors of HFMD transmission, such as virus subtypes and other infectious diseases, were not considered in our study due to data limitations^36^.

## Methods

### Ethics declarations

Our study was approved by the institutional review board of the School of Public Health, Sichuan University. All HFMD surveillance data were collected from the China Information System for Disease Control and Prevention. The study methods were carried out in accordance with relevant guidelines and regulations. Our study was constructed at the population level. Since all of the patients’ records were anonymized and no individual information could be identified, informed consent was not required.

### Study region

**Figure 6:**
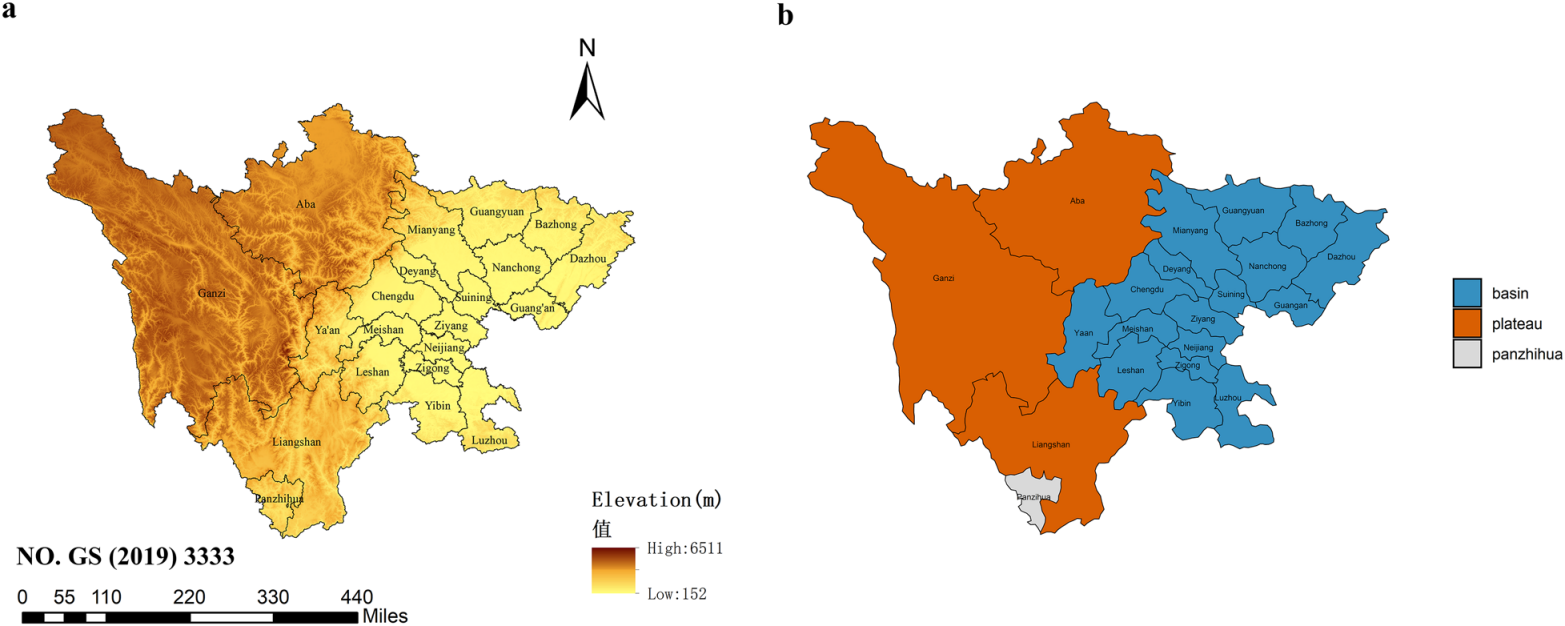
(**a**) Elevation of Sichuan Province; (**b**) Basin and plateau regions in Sichuan Province. The original map image was obtained from the China Ministry of Natural Resources (http://bzdt.ch.mnr.gov.cn/index.html).

Sichuan Province is an inland province in Southwest China (26.40°N–33.68°N and 98.31°E–107.99°E) with a population of 8.3 million (in 2017). Sichuan Province consists of 21 cities covering an area of 486 052 km^2^, and the city-specific socioeconomic characteristics differ. Surrounded by the plateau region in the west, Sichuan Province has a complex terrain (Fig. 6a)^23^. Due to the differences in the terrain and socioeconomic conditions, there are notable differences in the levels and seasonal trends of air pollutants between the basin region and the plateau region in Sichuan (Fig. 6b).

### Data sources

Daily HFMD case numbers in Sichuan Province from 2015 to 2017 were obtained from the China Information System for Disease Control and Prevention. Considering that over 99% of the patients with reported HFMD in the study were aged 0–14 years, only cases in those aged 0–14 years were included.

Air pollution variables, including hourly data of particulate matter (PM_2.5_ and PM_10_), nitrogen dioxide (NO_2_), carbon monoxide (CO), ozone (O_3_), and sulfur dioxide (SO_2_) for each city were collected from the China National Environmental Monitoring Center (http://www.cnemc.cn/). The daily mean concentration of each air pollutant was obtained by calculating the arithmetic mean of the hourly data. Meteorological data for the same period, including mean temperature and mean relative humidity, were provided by the China Meteorological Date Sharing Service System. Daily meteorological data for each city was obtained from 41 meteorological stations by ordinary kriging interpolation^37^. The missing values of air pollutants and meteorological variables were filled by linear interpolation^38^. Since the proportion of missing data was very small (< 1%), its impact on the results can be ignored.

### Statistical analysis

By combining the spatiotemporal Bayesian hierarchical model and the DLNM, we assessed the exposure–response and lag-response associations of air pollutants with HFMD to reveal exposure-lag-response relationships after accounting for spatiotemporal effects and observed confounders. A Negative binomial distribution was assumed for daily HFMD cases, and the model was constructed as follows:

1 $${Y}_{it}\sim NegBin({E}_{it} \cdot {\rho }_{it}, \kappa )$$

2 $$\mathrm{log}\left({\rho }_{it}\right)=\alpha +cb\left({M}_{itj},{df}_{v};L,{df}_{l}\right)+\sum ns({X}_{it},df)+{\upsilon }_{i}+{\nu }_{i}+{\gamma }_{t}$$

where ${E}_{it}$ and ${\rho }_{it}$ are the expected number of HFMD cases and relative risk (RR) of HFMD on calendar day $t(t=\mathrm{1,2},\dots 1096)$ in city $i(i=\mathrm{1,2},\dots 21)$, respectively; κ is the overdispersion parameter; $\alpha$ is the intercept; ${M}_{itj}$ is the air pollutant of interest $(j=\mathrm{1,2},\dots 6)$ on calendar day $t$ in city $i$; $cb\left(.\right)$ is a cross-basis function consisting of two natural cubic splines representing the lag- and exposure–response associations, respectively, and the degrees of freedom(${df}_{v}$ and ${df}_{l}$) for these spline functions were chosen by sensitivity analysis. The maximum lag period of 14 days ($L=14$) was selected based on previous studies and the incubation time before cases were reported^15,39^. The spatial effect used a Besag-York-Mollie model, thus the spatial structured random effect ${\upsilon }_{i}$ was specified as an intrinsic conditional autoregressive process and the spatial unstructured random effect ${\nu }_{i}$ was specified as an exchangeable prior: ${\nu}_{i}\sim Normal(0,{\sigma }_{\nu}^{2})$; ${\gamma }_{t}$ is the temporally structured effect to capture seasonal and long-term trends^40^; and ${X}_{it}$ denotes the other air pollutants and meteorological variables incorporated as confounding factors; a natural cubic spline with 3*df* and 14-day moving average were set to these confounders. There were six models for the six air pollutants, as only one cross-basis was included in each model to analyze the exposure-lag-response association between the air pollutant and HFMD. Since the RV-coefficient between PM_2.5_ and PM_10_ is 0.87 and 0.81 (Supplementary Table S3) in the cross-basis function and the natural cubic spline function, respectively. When analyzing the relationship between PM_2.5_ (PM_10_) and HFMD, the PM_10_ (PM_2.5_) were not included in the model as a confounder.

We conducted a stratified analysis by regions, the basin region consists of 17 cities, and the plateau region consists of 3 cities (Fig. 6b). The adjacency-based spatial weighted matrix (${w}_{ij}$ in the adjacency matrix is equal to 1 if city i and j are adjacent, but equal to 0 otherwise) in the two subregions was reconstructed based on two different map objects. For instance, in the 21 cities analysis, Aba has five adjacent cities according to the adjacency-based matrix, but there is only one adjacent city in the plateau analysis.

The posterior distributions of these parameters were estimated by Integrated Nested Laplace Approximation (INLA)^41^. We used the deviance information criterion (DIC) to measure the fit of the model; a smaller DIC value indicates a better fitted model^42^. The sensitivity analyses were performed considering the following: (1) A time smoother function (a natural cubic spline with 8 *df* per year) to control the temporal autocorrelations; and (2) the *df* of the natural cubic spline for both exposure response and lag response were 3–7. All analyses were conducted in R version 4.0.3 with the packages “*dlnm*”, “*splines*”, and “*INLA*”.

## Conclusion

In conclusion, our study assessed the exposure-lag-response associations of air pollutants with HFMD in 21 cities in Sichuan Province, with adjustments for spatiotemporal effects. The results indicated significant associations between PM_2.5_, PM_10_ and NO_2_ concentrations and HFMD, but no significant associations were observed between CO, O_3_, and SO_2_ concentrations and HFMD. These findings deepen the understanding of the associations between air pollutants and HFMD and provide evidence supporting the formulation of relevant prevention measures and the establishment of an early warning system.

## Supplementary Information

Supplementary Information.

## Data Availability

The datasets generated and/or analyzed during the current study are available from the corresponding author on reasonable request.
