# Supplementary material for: Associations between ambient air pollutants and childhood hand, foot, and mouth disease in Sichuan, China: a spatiotemporal study
Source: Sci Rep. 2023 Mar 10;13:3993. doi: 10.1038/s41598-023-31035-7 (PMC10006415; doi:10.1038/s41598-023-31035-7)
Supplement: Supplementary file 1 — Supplementary Information. [file 41598_2023_31035_MOESM1_ESM.docx]

**Associations between ambient air pollutants and childhood hand, foot, and mouth disease in Sichuan, China: A spatiotemporal study**

Jian Qian^1¶^, Caiying Luo^1¶^, Qiang Lv ^2^, Yaqiong Liu^2^, Tao Zhang^1^, Fei Yin^1^, Yue Ma^1^*, Tiejun Shui^3^*

1 West China School of Public Health and West China Fourth Hospital, Sichuan University, Chengdu, Sichuan, China

2 Sichuan Center for Disease Control and Prevention, Chengdu, Sichuan, China

3 Yunnan Center for Disease Control and Prevention, Kunming, Yunnan, China

¶: These authors contributed equally to this work.

*: Author for correspondence:

Yue Ma (Email: [gordonrozen@qq.com](mailto:gordonrozen@qq.com)),

Tiejun Shui (Email: 67637539@qq.com)

Supplementary Table S1. The DIC values for the six air pollutants when ${df}_{l}$are 3-7

| Air pollutant | ${df}_{l}$=3 | ${df}_{l}$=4 | ${df}_{l}$=5 | ${df}_{l}$=6 | ${df}_{l}$=7 |
| --- | --- | --- | --- | --- | --- |
| SO_2_ | 101595.4 | 101694.3 | 101685.6 | 101678.5 | 101663.9 |
| NO_2_ | 101582.3 | 101679.1 | 101668.4 | 101664.1 | 101658.0 |
| PM_10_ | 101536.7 | 101643.1 | 101614.8 | 101609.6 | 101601.5 |
| CO | 101579.5 | 101681.8 | 101647.6 | 101620.0 | 101608.8 |
| O_3_ | 101612.4 | 101678.4 | 101676.2 | 101678.5 | 101673.3 |
| PM_2.5_ | 101536.7 | 101647.1 | 101617.3 | 101607.8 | 101591.3 |

Supplementary Table S2. The DIC values for the six air pollutants when ${df}_{v}$are 3-7

| Air pollutant | ${df}_{v}$=3 | ${df}_{v}$=4 | ${df}_{v}$=5 | ${df}_{v}$=6 | ${df}_{v}$=7 |
| --- | --- | --- | --- | --- | --- |
| SO_2_ | 101595.4 | 101599.4 | 101600.9 | 101605.3 | 101608.3 |
| NO_2_ | 101582.3 | 101586.5 | 101585.2 | 101587.8 | 101594.6 |
| PM_10_ | 101536.7 | 101539.5 | 101539.9 | 101545.4 | 101542.8 |
| CO | 101579.5 | 101580.2 | 101584.1 | 101594.3 | 101592.0 |
| O_3_ | 101612.4 | 101611.4 | 101618.0 | 101620.9 | 101619.9 |
| PM_2.5_ | 101536.7 | 101531.8 | 101534.6 | 101538.0 | 101540.7 |

Supplementary Table S3. The RV-coefficient between PM_2.5_ and PM_10_ in cross-basis and natural cubic spline

| City | Cross-basis | Natural cubic spline |
| --- | --- | --- |
| Chengdu | 0.94 | 0.90 |
| Zigong | 0.97 | 0.94 |
| Panzhihua | 0.91 | 0.82 |
| Luzhou | 0.92 | 0.88 |
| Deyang | 0.94 | 0.86 |
| Mianyang | 0.93 | 0.83 |
| Guangyuan | 0.55 | 0.44 |
| Suining | 0.86 | 0.78 |
| Neijiang | 0.94 | 0.89 |
| Leshan | 0.96 | 0.91 |
| Nanchong | 0.94 | 0.90 |
| Meishan | 0.91 | 0.85 |
| Yibin | 0.95 | 0.91 |
| Guangan | 0.86 | 0.80 |
| Dazhou | 0.93 | 0.89 |
| Yaan | 0.90 | 0.85 |
| Bazhong | 0.93 | 0.87 |
| Ziyang | 0.84 | 0.77 |
| Aba | 0.61 | 0.43 |
| Ganzi | 0.63 | 0.55 |
| Liangshan | 0.91 | 0.88 |
| Mean | 0.87 | 0.81 |


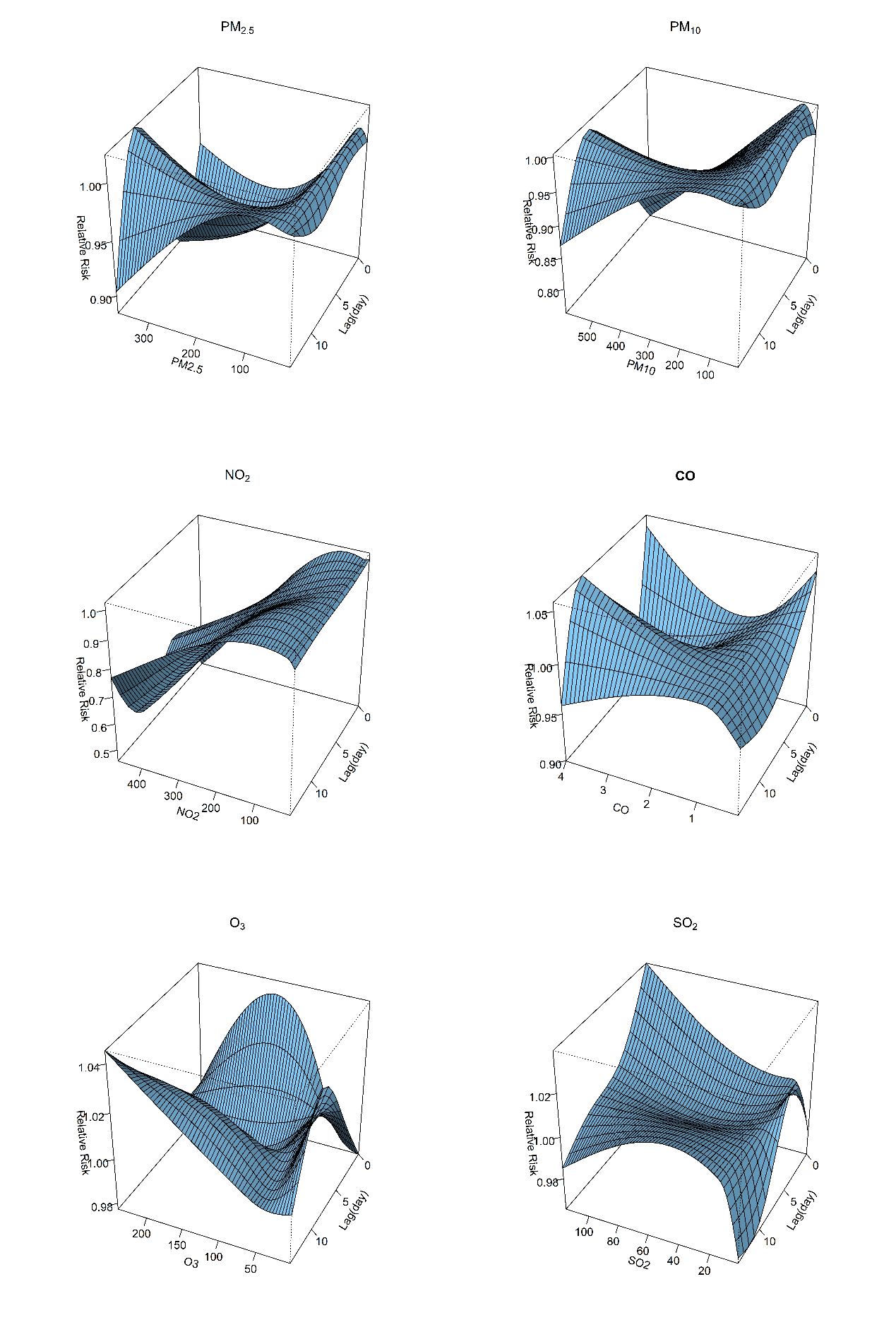


Supplementary Fig S1. Relationships between air pollutants and HFMD over 14 lag days


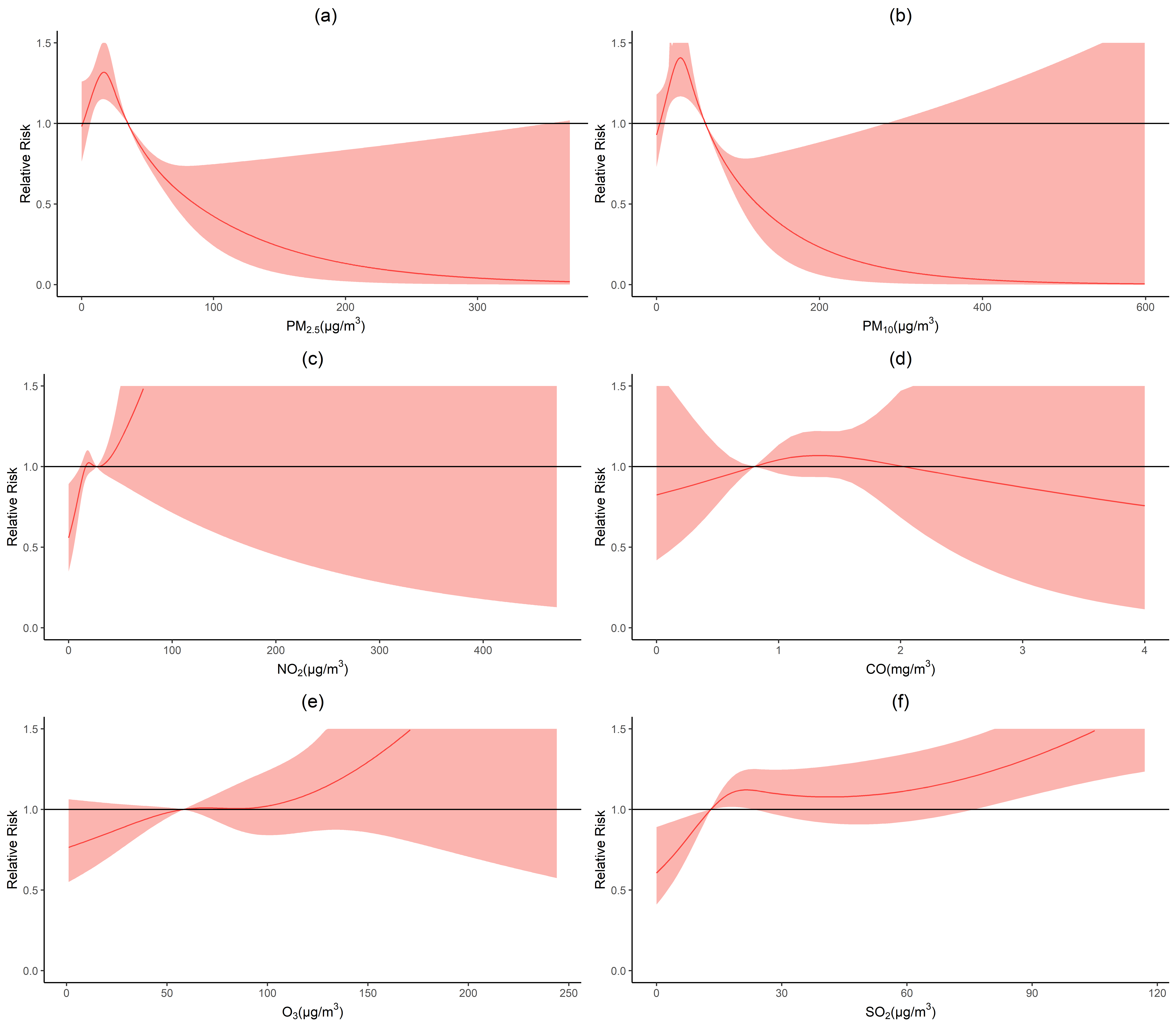


Supplementary Fig S2. The overall cumulative-response associations between air pollutants and HFMD of the two-stage DLNMs


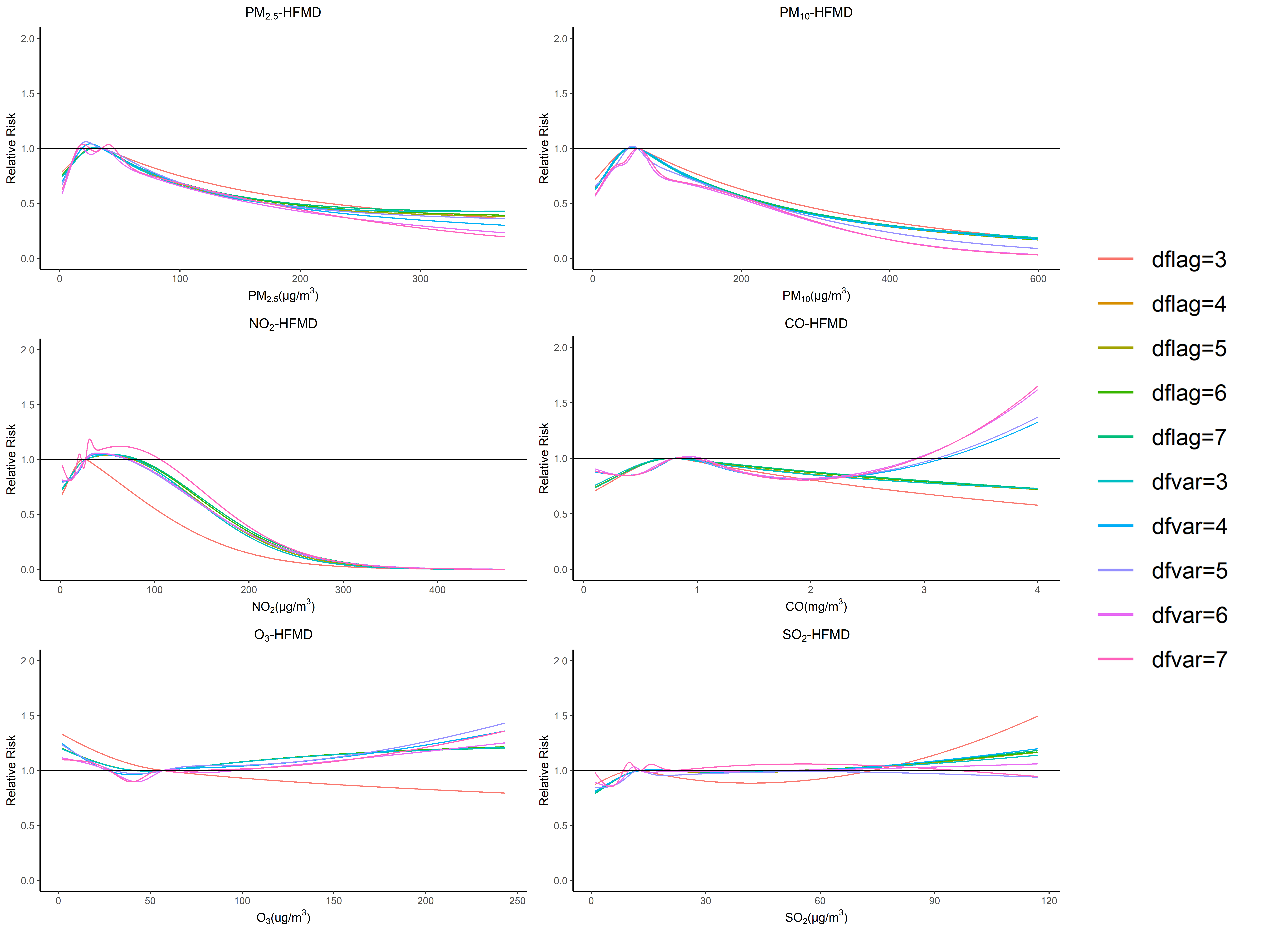


Supplementary Fig S3. Results of sensitivity analysis to the pre-specified parameters


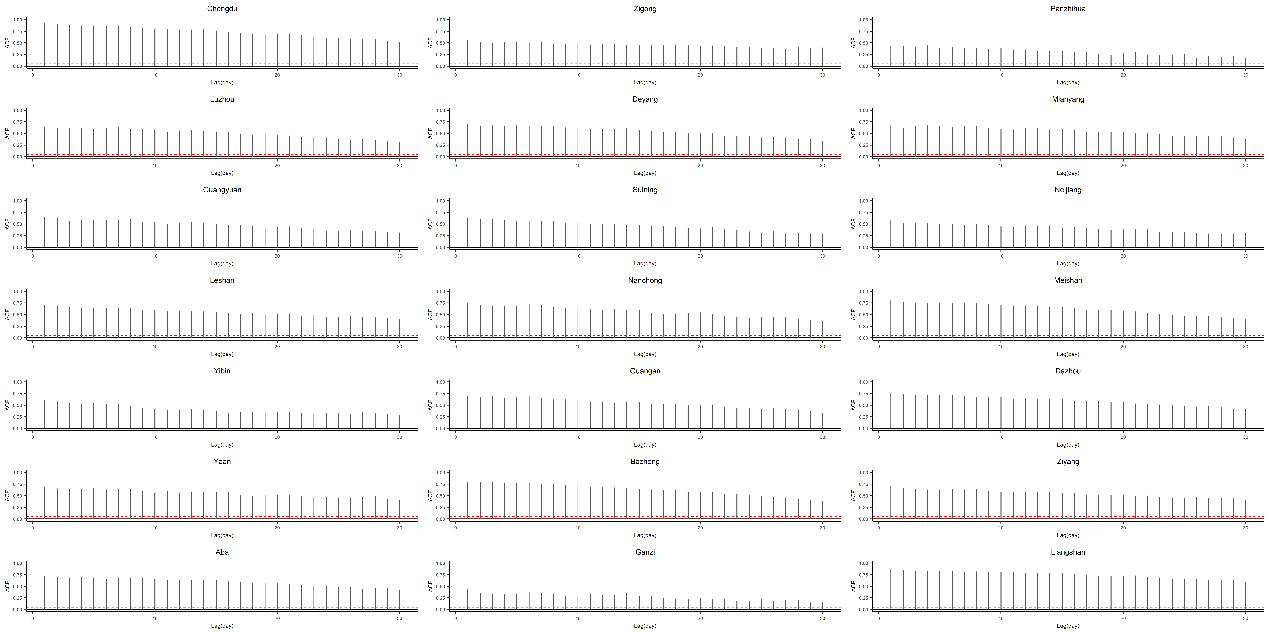


Supplementary Fig S4. ACF plot of the relative risk of HFMD in 21 cities


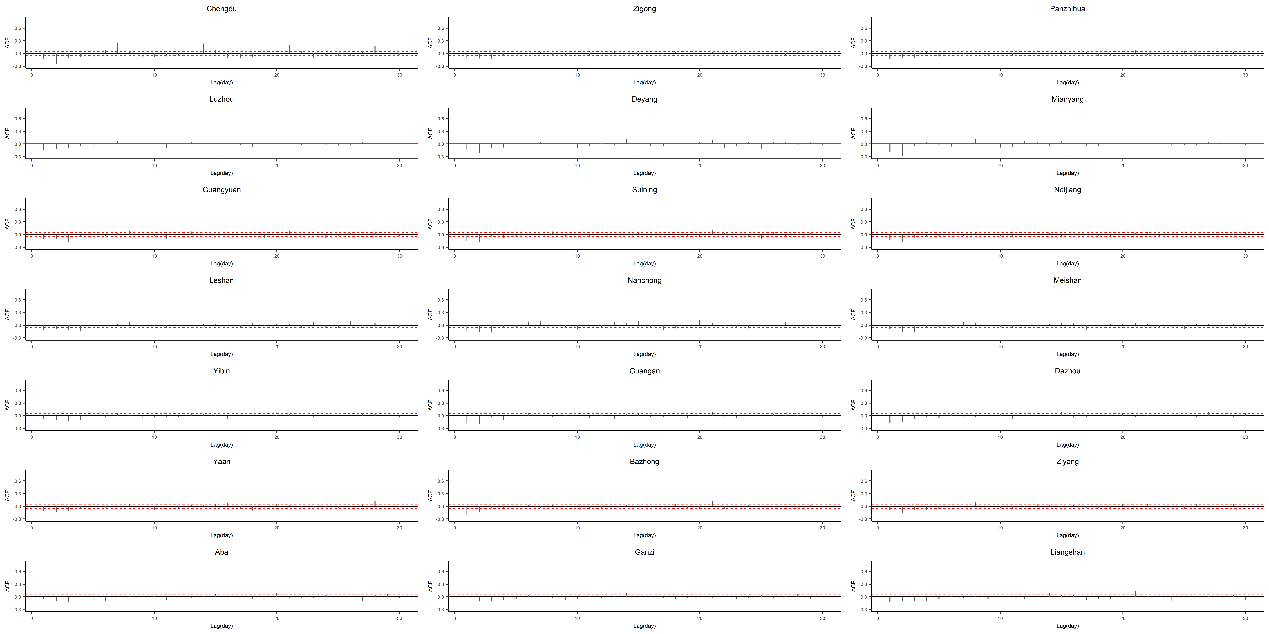


Supplementary Fig S5. ACF plot of the residual in 21 cities with temporal random effect


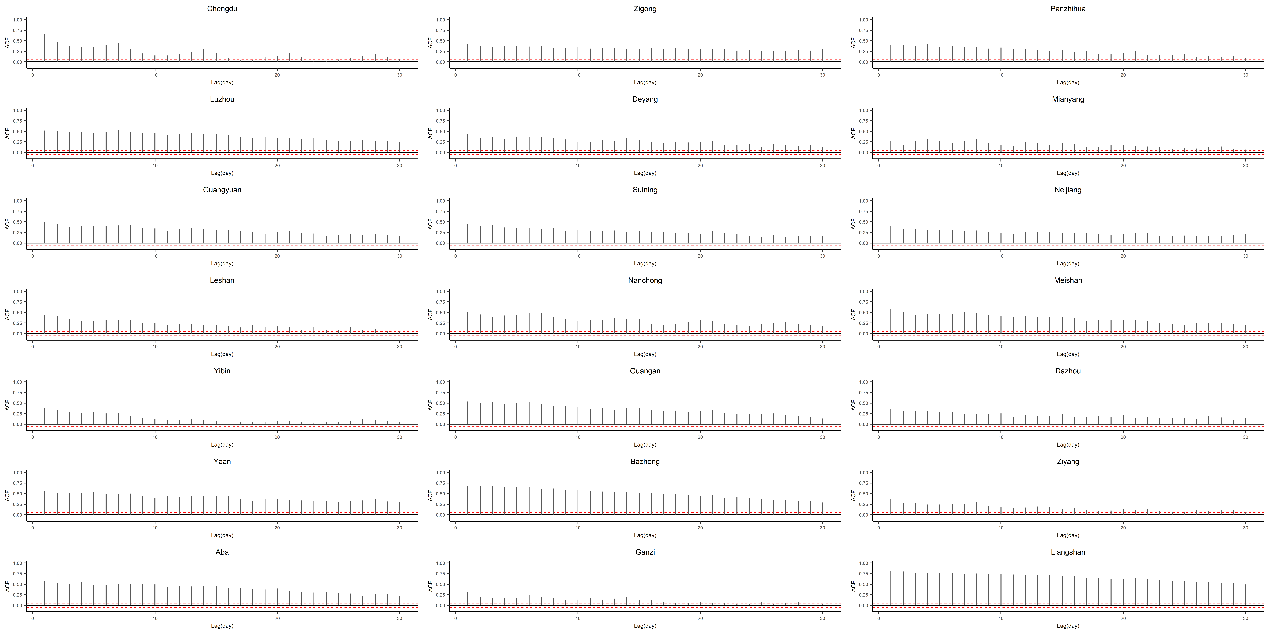


Supplementary Fig S6. ACF plot of the residual in 21 cities with a natural cubie spline function
